# An Overlapping Multi-Signal Detection Framework Based on Masked Pretraining and Repulsion Loss

**DOI:** 10.3390/s26206423

**Published:** 2026-10-11

**Authors:** Jiahao Ren, Xing Zeng, Xikang Wang, Hua Xu

**Affiliations:** Information and Navigation School, Air Force Engineering University, Xi’an 710077, China; 15602018733@163.com (J.R.); wangxkmaki@163.com (X.W.); xu.hua@139.com (H.X.)

**Keywords:** overlapping signal detection, self-supervised pretraining, Swin-Transformer, repulsion loss

## Abstract

As the electromagnetic environment grows increasingly crowded with coexisting wireless systems, overlapping signals have become commonplace. Signal overlap in a limited space leads to feature mixing, boundary blurring, and annotation difficulty, which existing deep learning methods cannot effectively handle. To tackle these challenges, we propose a two–stage detection framework that combines SimMIM self-supervised pretraining with a repulsion loss. In the pretraining phase, a Swin-Tiny backbone is trained on unlabeled time–frequency images through masked reconstruction. In the fine-tuning phase, the backbone is transferred to a YOLOv8 detector, where the repulsion loss is optimized jointly with the base detection loss. In the component ablation, the complete framework improves mAP50 by 9.0 points over the random-initialized baseline on the validation set. On the held-out main and overlap-stress sets, it obtains 0.875 mAP50, 0.798 mAP50-95, and 0.873 mAR, and 0.838, 0.756, and 0.829, respectively. Compared with the YOLOv8 baseline, the observed mAP50 gains are 1.95 and 2.18 points, respectively. The main high-overlap subset is small; its observed 1.64-point margin is therefore reported as descriptive evidence. These results indicate that in-domain self-supervised pretraining combined with spatially constrained box regression provides an effective representation and detection strategy for overlapping signals.

## 1. Introduction

The electromagnetic spectrum is a core strategic resource for both wireless communications and electronic warfare [1]. Recently, the widespread deployment of 5G networks and the Internet of Things has intensified the time–domain and frequency–domain overlap of multi-system signals, making dense signal overlap the norm in modern radio-frequency (RF) environments [2]. Accurate detection and identification of overlapping signals are therefore fundamental to spectrum awareness and electromagnetic situation assessment.

Traditional signal detection methods, such as energy detection and cyclostationary feature analysis, rely on handcrafted features and fixed decision thresholds [3,4]. Although computationally efficient, they depend heavily on prior signal knowledge and cannot reliably separate overlapping signal components or delineate their time–frequency boundaries, falling short of the requirements for precise spectrum management in complex environments.

Advances in computer vision have enabled a powerful paradigm for intelligent RF signal processing: radio signals are converted into time–frequency spectrum via the Short-Time Fourier Transform (STFT) and then processed by object detection models [5]. Studies have shown that such methods outperform traditional approaches in conventional non-overlapping scenarios [3,5].

However, in dense overlapping signal scenarios, existing methods still face significant challenges. First, signal overlap leads to feature mixing, which can easily mislead the detector into assigning features to the wrong predicted boxes and producing false detections. In addition, overlap patterns are highly diverse in terms of location, scale, and aspect ratio, requiring the network to learn a wide variety of overlap patterns from a large number of samples; traditional deep learning methods struggle to effectively extract discriminative signal features. Second, standard detection loss functions only constrain the alignment between each predicted box and its corresponding ground-truth box, ignoring the spatial relationships between adjacent boxes. This results in bounding box drift and severe overlap among predictions, thereby degrading localization accuracy and exacerbating missed detections. Third, annotating overlapping signals is extremely costly and difficult. Compared with natural images, signal boundaries in spectrograms are inherently ambiguous, making manual annotation of the time–frequency boundaries of individual signals time-consuming and laborious. Overlapping further blurs the target boundaries, rendering precise annotation almost infeasible and leading to an acute scarcity of publicly available benchmark datasets for overlapping signals.

To address these challenges, we propose a framework for overlapping signal detection that integrates two previously independent research lines: self-supervised representation learning and spatially constrained bounding-box regression. In the pretraining stage, SimMIM [6] is deployed on a Swin-Transformer backbone to learn effective time–frequency features from a large corpus of unlabeled spectrograms. In the fine-tuning stage, the pretrained backbone is integrated into a YOLOv8 detection framework, and repulsion loss [7] imposes explicit spatial constraints on bounding boxes, alleviating prediction box adhesion and localization drift in overlapping regions.

The main contributions of this paper are summarized as follows:

- To address the local feature ambiguity caused by the feature mixing of overlapping signals, we adopt the Swin-Transformer backbone with window self-attention and shifted windows, which establishes cross-window global feature modeling at linear computational complexity, improving the discrimination of signals in overlapping scenarios.
- To overcome the high labeling cost of modulation signal datasets, we adopt SimMIM self-supervised pretraining: the backbone learns effective feature representations from a large number of unlabeled time–frequency images in the same domain, which not only avoids extra annotation but also improves the feature extraction capability of the downstream detector through backbone transfer during fine-tuning.
- To adapt the repulsion loss to overlapping RF signals, we modify the original IoG-based RepGT into a gated IoU-excess formulation that subtracts the natural overlap between matched and interfering ground-truth boxes.
- To fully validate the effectiveness of the proposed pretraining framework and the repulsion loss, we perform experiments in the pretraining and fine-tuning stages. Compared with the YOLOv8 baseline, the proposed method achieves the best mAP50 of 0.862 (mAP50-95 0.778, mAR 0.866) in the overlapping-signal scenario, outperforming YOLOv8 by 2.9 points in mAP50, 3.7 points in mAP50-95, and 4.2 points in mAR. And we find the largest differences occur in the more difficult regimes: 7.4 mAP50 points at the lowest SNR bin and 6.4 points on the validation high-overlap tier (paired-bootstrap estimate 6.26 points, 95% CI [+2.63, +10.02]).

## 2. Related Work

### 2.1. Feature Extraction and Pretraining

In the STFT-based time–frequency spectrogram detection task, the backbone network is responsible for extracting signal feature representations from the spectrogram, and its architectural design directly determines the quality of feature extraction under overlapping conditions. Early works adopted CNNs such as ResNet and DarkNet as the backbone [8,9]; however, the local receptive field of convolutions limits the modeling of signal dependencies across frequency bands and time intervals. To address these drawbacks, channel and spatial attention modules were introduced to highlight critical regions through dynamic weighting, but they merely recalibrate convolutional features rather than directly establishing associations between feature positions [10,11].

Unlike convolutions, which progressively expand their coverage through local receptive fields, self-attention establishes global dependencies directly between feature positions. The transformer architecture proposed by Vaswani et al. models global dependencies among sequence elements through self-attention [12], overcoming the limitation of local receptive fields in convolutions. However, the computational complexity of standard self-attention grows quadratically with input size, making it difficult to apply directly to high-resolution images. Liu et al. introduced self-attention into the vision domain with Swin-Transformer [13], which adopts a hierarchical structure and shifted windows: self-attention is computed within windows to control complexity, while the shifted window mechanism alternates window partitions between consecutive layers so that cross-window information is progressively exchanged through multi-layer propagation. This design offers a balance between global receptive fields and computational feasibility, and its hierarchical features are naturally suited to multi-scale object detection. Building on this, researchers introduced Swin-Transformer to RF signal detection and recognition, demonstrating the feasibility of window-based self-attention for time–frequency spectrogram modeling [14]. Furthermore, some researchers have adopted Swin-Transformer-based instance segmentation networks to separate individual signal components from overlapping multi-component signals whose features are interwoven in the time–frequency image [15]. In practice, structural parameters such as the window size are usually adapted to the signal’s time–frequency characteristics to fully exploit the modeling capability for wideband signals.

However, the backbone methods above all rely on supervised training with large-scale labeled data. In general vision, this requirement is met by public benchmarks such as ImageNet. For overlapping signals, labels at that scale are not available and are costly to produce; self-supervised pretraining on unlabeled data has therefore become an important path for alleviating this annotation bottleneck [2].

In self-supervised pretraining, contrastive learning was an early direction. Methods such as SimCLR and MoCo v2 construct positive pairs through data augmentation and pull paired instance representations closer in feature space [16], while BYOL and SimSiam further removed the dependency on negative samples [17,18]. These methods perform well in image-level classification tasks such as modulation recognition. Their feature representations, however, rely on global average pooling, which compresses the fine-grained spatial information required to localize each signal component in overlapping regions and conflicts with the spatial-resolution requirements of detection. This structural conflict motivates the exploration of masked image modeling (MIM) for time–frequency feature learning.

Masked image modeling (MIM) guides feature learning by randomly masking image regions and reconstructing the masked content from visible context, an idea originating from BERT’s masked language modeling in natural language processing [19]. Bao et al. first adapted it to vision through BEiT [20], and MAE, proposed by He et al., substantially lowered the barrier to MIM pretraining by combining a high masking ratio with an asymmetric encoder-decoder that reconstructs directly in pixel space [21]. It has also been applied to modulation recognition and signal classification in RF signal processing, and Zhao et al. introduced MAE to overlapping signal detection, achieving notable gains in few-shot settings [2]. In parallel, object detection networks such as Mask R-CNN [22] and density-guided YOLO have been directly applied to overlapping multi-signal recognition in time–frequency images [23]. However, MAE’s asymmetric design restricts the encoder to processing only the visible patches, which constitute approximately 25% of the total, leaving the decoder to shoulder the bulk of the reconstruction burden; the backbone’s encoding of fine-grained local spectrogram features during pretraining may therefore be insufficient. In contrast, SimMIM’s lightweight decoder shifts the reconstruction burden onto the encoder, encouraging the backbone to retain richer local spatial information needed for detection [6]. Nevertheless, the empirical effectiveness of SimMIM in RF overlapping signal detection still awaits systematic experimental validation.

### 2.2. Bounding-Box Regression Loss Functions

In RF overlapping signal detection, bounding-box regression losses adjust predicted box positions by minimizing the discrepancy between predicted boxes and their matched ground-truth boxes, and their design directly determines localization accuracy in overlapping scenarios [24]. The commonly used losses are SmoothL1 and the IoU loss. The IoU metric subsequently evolved into GIoU, DIoU, and CIoU, which respectively introduce the smallest enclosing box, center-distance, and aspect-ratio constraints to mitigate the lack of gradients when the predicted and ground-truth boxes do not overlap and to refine regression accuracy [25,26,27]. These variants were also adopted in time–frequency detection tasks, and a hybrid strategy that dynamically switches between DIoU and CIoU was developed to address the elongated morphology of wideband signals [28].

However, in dense overlapping scenarios, the ground-truth boxes of multiple signals are adjacent or even partially overlapping in the time–frequency plane; a predicted box inevitably overlaps with neighboring signals while aligning to its matched ground-truth box, making its assignment difficult to determine. This assignment ambiguity arises because the above losses consider only the alignment between a predicted box and its matched ground-truth box, without penalizing its overlap with the ground-truth boxes of neighboring signals.

In dense pedestrian detection, Wang et al. proposed repulsion loss to resolve this assignment ambiguity; it simultaneously requires each predicted box to approach its matched ground-truth box and to stay away from surrounding signals. Repulsion loss comprises two constraints: the repulsion ground-truth loss (RepGT) penalizes, via the IoG metric, the overlap between a predicted box and its nearest non-matched ground-truth box, suppressing drift toward neighboring signals; the predicted-box repulsion term (RepBox) imposes IoU-based mutual exclusion on predicted boxes matched with different ground-truth boxes, reducing the probability that competing detections are removed by non-maximum suppression (NMS) and making the detector less sensitive to the choice of NMS threshold [7]. Unlike post-processing schemes that act only at inference, such as soft-NMS, repulsion loss embeds spatial constraints into the training objective [29]. Overlap suppression is then optimized end-to-end with the network rather than relying on empirically tuned post-processing thresholds, thereby alleviating assignment ambiguity and reducing missed detections in overlapping scenarios. Repulsion loss, however, was designed for dense scenarios in natural images. Signals in spectrograms appear as continuous time–frequency regions whose geometry differs fundamentally from the compact, well-bounded objects in natural images. How repulsion loss should be adapted to the time–frequency region morphology of RF overlapping signals remains to be systematically explored.

## 3. System Model

In broadband electromagnetic sensing and communication reconnaissance scenarios, signals from multiple independent emitters are superimposed at the receiver after propagation through the channel. When the carrier frequencies of different signals are close, and their time–domain durations overlap, the received signal exhibits varying degrees of overlap in the time–frequency plane, making it difficult to detect individual signal components and accurately delineate their time–frequency boundaries, which poses significant challenges to the detection and identification of overlapping signals.

Consider independent emitters in space, whose transmitted RF signals are defined as:

(1) sk(t) = Rexk(t)ej2πfkt

Here, $x_{k}(t)=x_{I,k}(t)+jx_{Q,k}(t)$ is the complex baseband waveform after shaping filtering; $x_{I,k}(t)$ and $x_{Q,k}(t)$ are the in-phase and quadrature components, respectively; $f_{k}$ is the carrier frequency of the signal [30].

After receiving the RF signal, the receiver performs quadrature down-conversion and low-pass filtering to obtain the baseband received signal. Assuming N independent emitters exist within the observation bandwidth, the overlapping baseband received signal can be expressed as the superposition of multiple signal components and noise:

(2) $$y(t)=\sum_{k=1}^{N}H_{k}(t)x_{k}(t)e^{j2\pi \Delta f_{k}t}+n_{\mathrm{BB}}(t)$$

Here, $H_{k}(t)$ denotes the baseband channel response of the k signal, $\Delta f_{k}$ is its carrier-frequency offset, and $n_{BB}(t)$ is complex additive white Gaussian noise. For the dataset used in this study, all optional channel and receiver impairments in the TorchSig pipeline were disabled; therefore $H_{k}(t)=1$, and the received signal is the superposition of the transmitted signals plus AWGN.

When the frequency-offset intervals of multiple signals overlap, and their time–domain durations intersect, the signal components are aliased in the baseband time domain, ultimately manifesting as overlap in the time–frequency plane. To distinguish multiple signal components superimposed in the time domain, the Short-Time Fourier Transform (STFT) is adopted to map the one-dimensional baseband signal onto the two-dimensional time–frequency plane (spectrogram), so that different signal components can be distinguished through joint time–frequency features. For the discretely sampled baseband received signal, its discrete STFT is defined as [31]:

(3) $$S(m,f)=|Y(m,f){|}^{2}$$

Here, Y(m,f) denotes the windowed STFT of the received signal y(t) computed with a Blackman window of length 512 and a hop size of 512 (zero overlap).

Before feeding the time–frequency image into the target detection model, the STFT result is typically normalized with the infinity norm to eliminate amplitude differences among signals, and then the power spectrum is scaled in decibels to enhance the feature representation of low-energy signals for deep learning models.

Concretely, the infinity-norm normalization fixes the strongest time–frequency bin of each image at 0 dB, and the dB image is then mapped linearly onto the 8-bit intensity range of the stored image (strongest bin to level 255, weakest bin to level 0), with no additional clipping or fixed dynamic-range parameter. Over the released corpus, this corresponds to an effective dynamic range of 83 dB (median; interquartile range 76–89 dB), i.e., approximately 0.33 dB per intensity level, which is well below the 2 dB SNR bin width.

## 4. Method

Based on the characteristics of the overlapping signal detection task, the proposed method is designed at three levels, namely the backbone network, self-supervised pretraining, and the detection loss. First, a Swin-Transformer backbone based on window self-attention and shifted windows is adopted. Window attention precisely captures local time–frequency features, and shifted windows propagate information between adjacent windows, establishing cross-window global interactions at linear computational complexity. The hierarchical feature maps provide multi-scale features matching the large span of signal bandwidths, laying a global modeling foundation for distinguishing overlapping signals. Second, as the Swin-Transformer is compatible with masked-reconstruction self-supervised pretraining, SimMIM is adopted to pretrain the backbone on large-scale unlabeled time–frequency images from the same domain. This alleviates the scarcity of labeled data in overlapping signal detection and enables the backbone to learn feature representations robust to overlap. Finally, to address the missed detections and false detections caused by the mutual crowding of predicted boxes in overlapping scenarios, a repulsion loss is introduced. It constrains the spatial relationships between each predicted box and the ground-truth boxes other than its matched one, as well as between different predicted boxes. The three levels target feature mixing, label scarcity, and interference among predicted boxes, respectively, together forming an integrated framework for overlapping signal detection.

This section presents a method for overlapping signal detection based on SimMIM self-supervised pretraining. Section 4.1 presents the overall framework of the detection method; Section 4.2 elaborates the design of the SimMIM self-supervised pretraining module; Section 4.3 introduces the specific design of the overlapping signal detection network and the repulsion loss.

### 4.1. Overall Framework

**Figure 1:**
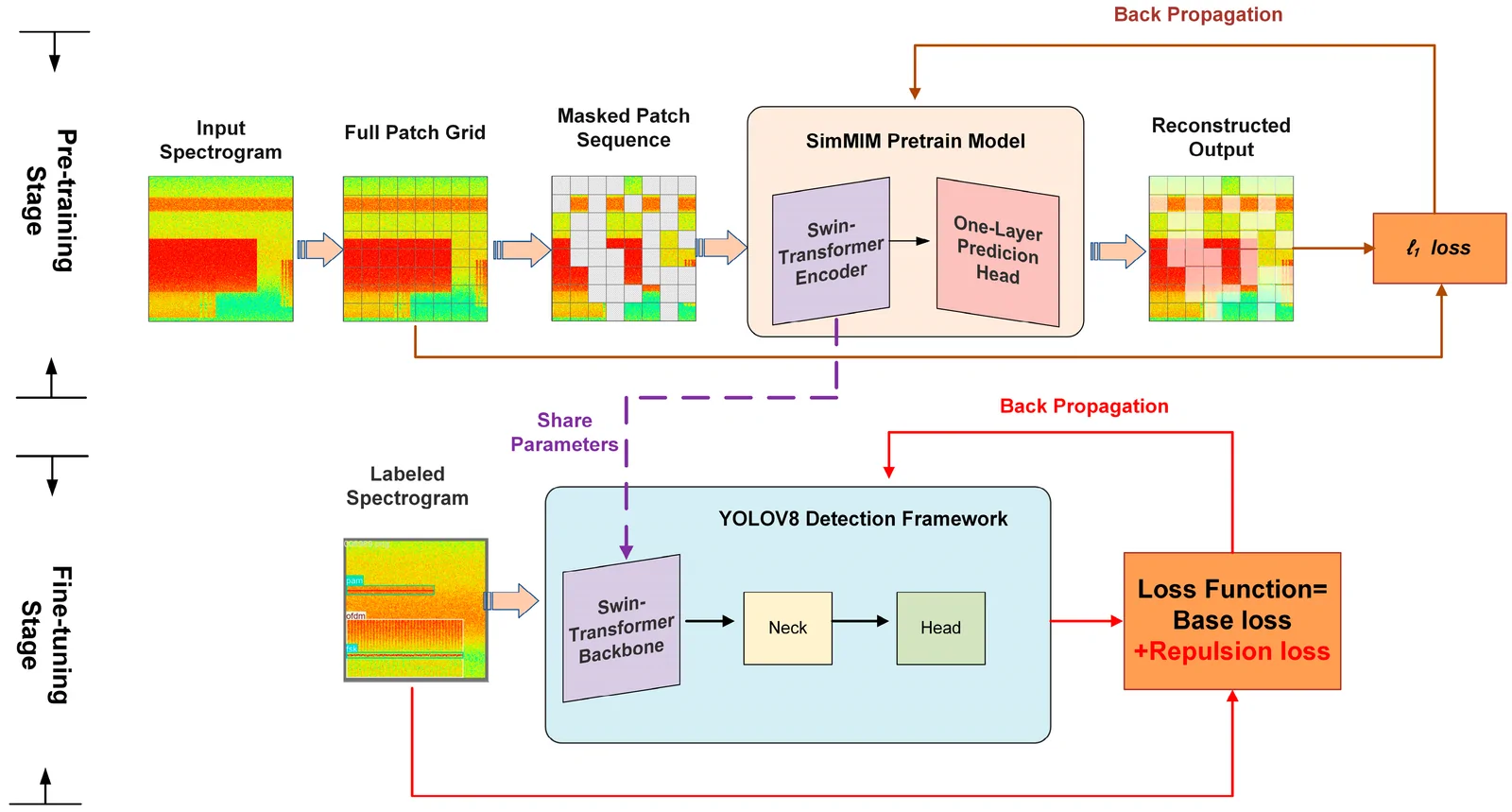
Overall architecture of the proposed method.

The overall pipeline of the method is shown in Figure 1, which consists of two stages: self-supervised pretraining and fine-tuning. In the pretraining stage, large-scale unlabeled time–frequency spectrograms are taken as input, and the Swin-Transformer backbone is trained through the SimMIM masked reconstruction task, enabling the backbone to acquire feature representations for overlapping time–frequency images. In the fine-tuning stage, the pretrained backbone weights are transferred to the YOLOv8 detection network, and the basic detection loss and the repulsion loss are jointly optimized on labeled overlapping signal data, where the repulsion loss imposes a spatial mutual-exclusion constraint on box regression to suppress the overlap between predicted boxes.

### 4.2. SimMIM Self-Supervised Pretraining

This paper adopts SimMIM for self-supervised pretraining on the Swin-Transformer backbone, motivated by two problems: the scarcity of labeled data in overlapping signal detection and the difficulty of extracting features from aliased signals. The pretraining process requires no manual annotation and utilizes only large-scale unlabeled time–frequency spectrograms. The SimMIM pretraining pipeline consists of three steps, namely masking, encoding, and reconstruction, whose specific design is given below.

Before pretraining, all spectrograms are upsampled from 512 × 512 to 640 × 640, matching the input resolution of the downstream detector and avoiding a resolution mismatch during backbone transfer. The input time–frequency spectrogram is first divided into 4 × 4 patches for embedding, and then a 20 × 20 mask grid is formed with 32 × 32 mask units corresponding to roughly one-quarter of a typical signal time–frequency footprint. Mask units are randomly selected for masking with a masking ratio of 60%, and the patch embeddings corresponding to the masked units are replaced by a learnable mask token. A 60% masking ratio follows the recommended SimMIM setting [19] and is lower than the 75% used in MAE. This ratio is appropriate because the encoder processes the complete token sequence and therefore retains richer visible context; at the same time, the visible region is insufficient to recover the masked time–frequency structure directly, which encourages the backbone to learn local time–frequency details such as signal boundaries and occupancy.

The masked token sequence is fed into the encoder for feature encoding. The encoder adopts the Swin-Transformer backbone, which transfers information between adjacent windows through window self-attention and shifted window mechanisms, gradually establishing cross-window long-range interactions over multiple layers while maintaining linear computational complexity; its local-window processing matches the locally concentrated distribution of signal time–frequency regions. The Swin-Tiny backbone used in this paper has about 28 M parameters and a window size of 10, set to match the typical time and frequency spans of signal time–frequency regions; following the official Swin implementation, feature maps are padded to multiples of the window size before window partitioning and cropped back afterward, so non-divisible resolutions are handled by cyclic padding without additional parameters. Unlike MAE, which encodes only visible patches, SimMIM replaces the embeddings at masked positions with mask tokens and performs self-attention over the complete token sequence.

The features output by the encoder are fed into a single-layer linear decoder to reconstruct the masked pixels. The decoder consists of a 1 × 1 convolutional layer and pixel shuffle, which maps the patch features to pixel values and restores the full-resolution image of 640 × 640. The reconstruction loss is computed only in the masked region, and the L1 loss is adopted to measure the difference between the reconstructed pixels and the original pixels:

(4) $$L_{\mathrm{simmim}}=(\frac{1}{M})\sum_{i\in \Omega }\left|x_{i}-{\hat{x}}_{i}\right|$$

Here, Ω is the set of masked pixels, M is its size, $x_{i}$ is the original pixel value, and ${\hat{x}}_{i}$ is the reconstructed pixel value.

**Figure 2:**
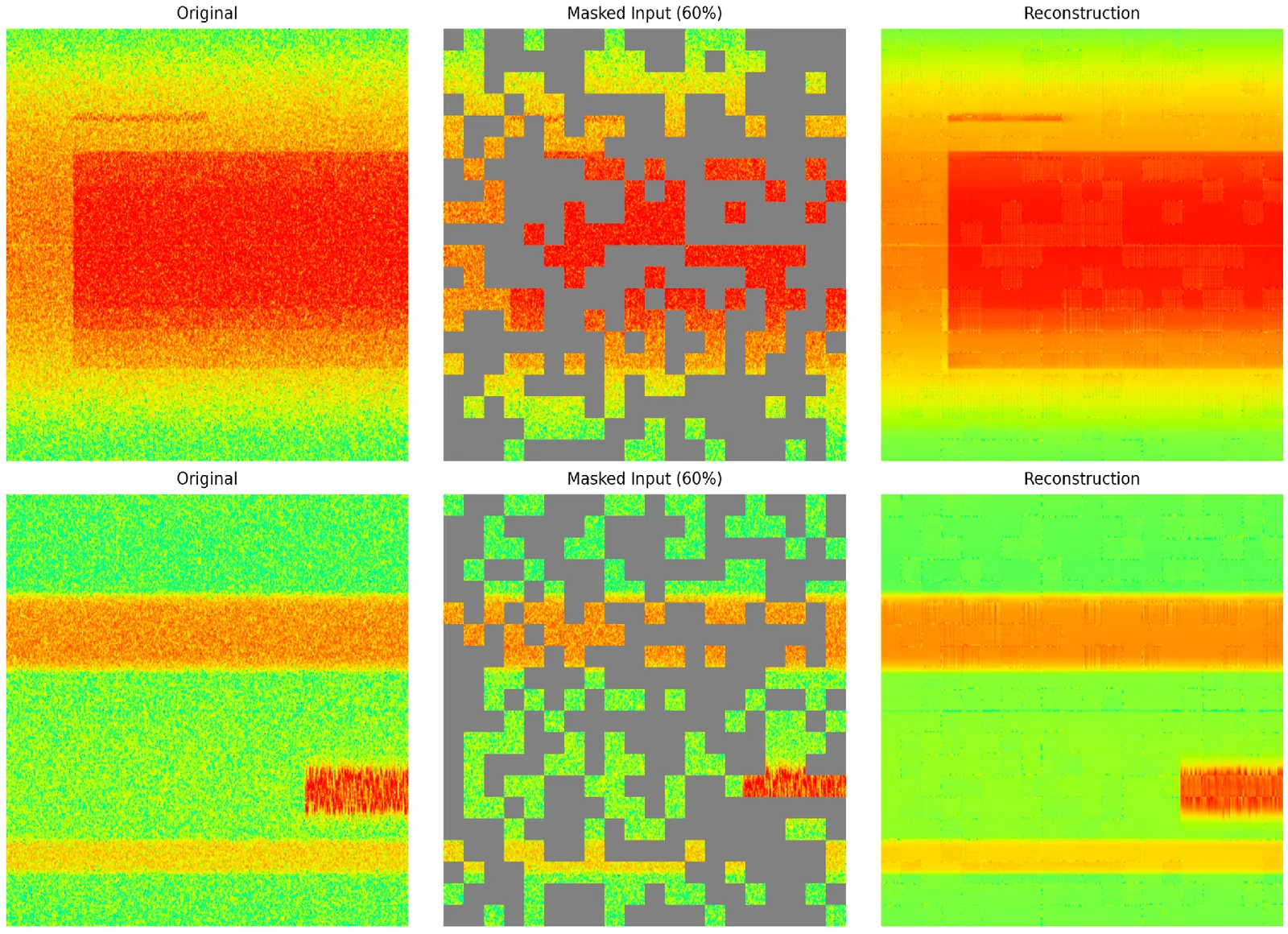
Mask-and-reconstruction examples of SimMIM pretraining.

The mask-and-reconstruction examples of SimMIM pretraining are shown in Figure 2.Through the above masked reconstruction pipeline, the backbone learns time–frequency features that remain discriminative under overlap. Overlapping signals partially cover one another in the time–frequency plane, so the visible parts alone are insufficient to distinguish individual signals. Random masking training addresses precisely this difficulty: the backbone is required to reconstruct the masked structure from the visible context, thereby acquiring the ability to infer the whole from parts; as a result, during downstream feature extraction, a partially covered time–frequency region can still elicit a complete feature response. The pixel-level reconstruction objective requires the backbone to preserve spatial details such as the boundaries and positions of time–frequency regions, directly supporting bounding box localization. Since the pretraining and downstream detection are conducted in the same time–frequency image domain, the learned features can be directly transferred to the detection network.

### 4.3. Detection Network for Overlapping Signals and Repulsion Loss

In the fine-tuning stage, the pretrained backbone weights serve as the initial values of the YOLOv8 backbone. The feature maps output by the 2nd, 3rd, and 4th stages of the backbone have strides of 8, 16, and 32, respectively, preserving time–frequency region information at different scales, and are fed into the neck network after channel alignment via convolutions. The neck adopts a PAN-FPN structure: the top-down path fuses high-level semantics, and the bottom-up path supplements low-level spatial details, enabling features at all scales to possess both semantic discrimination and localization ability [32]. The detection head adopts the YOLOv8 anchor-free decoupled structure, with a classification branch and a regression branch operating in parallel. At three scales, they output the per-position confidence for each category and the bounding box offsets, respectively, which are decoded and post-processed by non-maximum suppression (NMS) to obtain the final detection results for each time–frequency region.

The detection network is supervised by a basic detection loss. The basic detection loss consists of a classification loss and a bounding box regression loss. The classification loss adopts binary cross-entropy (BCE) to measure, category by category, the difference between the prediction and the label:

(5) $$L_{cls}=-\left(\frac{1}{N_{pos}}\right)\sum_{c=1}^{C}\sum_{i=1}^{N_{pos}}\left[y_{ic}\log{\hat{y}}_{ic}+\left(1-y_{ic}\right)\log\left(1-{\hat{y}}_{ic}\right)\right]$$

Here, $N_{pos}$ is the number of positive samples predicted boxes, $y_{ic}$ is the ground-truth label of the i predicted box on category c, and ${\hat{y}}_{ic}$ is the corresponding predicted probability.

The bounding-box regression loss $L_{\mathrm{box}}$ is the Complete IoU (CIoU) loss, while the boundary distribution is supervised separately by the Distribution Focal Loss [33]:

(6) $$L_{box}=L_{CIoU}$$

CIoU incorporates center-point distance and aspect-ratio consistency constraints on the basis of IoU:

(7) $$L_{CIoU}=1-IoU+\frac{\rho^{2}(b,b_{gt})}{c^{2}}+\alpha v$$

Here, ρ is the Euclidean distance between the center points of the predicted box and the ground-truth box, c is the diagonal length of the smallest enclosing rectangle, v and α measure and weight the aspect-ratio consistency, respectively.

DFL models the boundary distance as a discrete probability distribution, focusing the distribution of the predicted box boundary over discrete sampling points near the true value:

(8) $$L_{DFL}=-\left[\left(y_{i+1}-y\right)\log{\hat{y}}_{i}+\left(y-y_{i}\right)\log{\hat{y}}_{i+1}\right]$$

Here, y is the true value of the boundary distance, $y_{i}$ and $y_{i+1}$ are its adjacent discrete sampling points, and ${\hat{y}}_{i}$ and ${\hat{y}}_{i+1}$ are the corresponding predicted probabilities.

The basic detection loss works effectively in simple scenarios. However, when multiple signals overlap densely on the time–frequency image, the ground-truth boxes of these signals are highly adjacent or even partially overlapping in space, and the basic detection loss cannot guarantee detection accuracy. On the one hand, the basic detection loss only constrains each predicted box to move toward its corresponding ground-truth box, without constraining the relationship among predicted boxes. In dense overlapping scenarios, multiple candidate predicted boxes may simultaneously point to the same ground-truth box, while adjacent ground-truth boxes are likely to be missed. The time–frequency regions of overlapping signals are interwoven, and the boundaries of adjacent targets are difficult to define precisely, so predicted boxes tend to overlap at blurred boundaries. On the other hand, although the CIoU loss introduces center-point distance and aspect-ratio consistency constraints, a predicted box moving toward a non-corresponding ground-truth box is not penalized when two ground-truth boxes are closely adjacent. It may even receive erroneous gradient guidance from the center-distance term of CIoU, further aggravating the overlap of predicted boxes.

**Figure 3:**
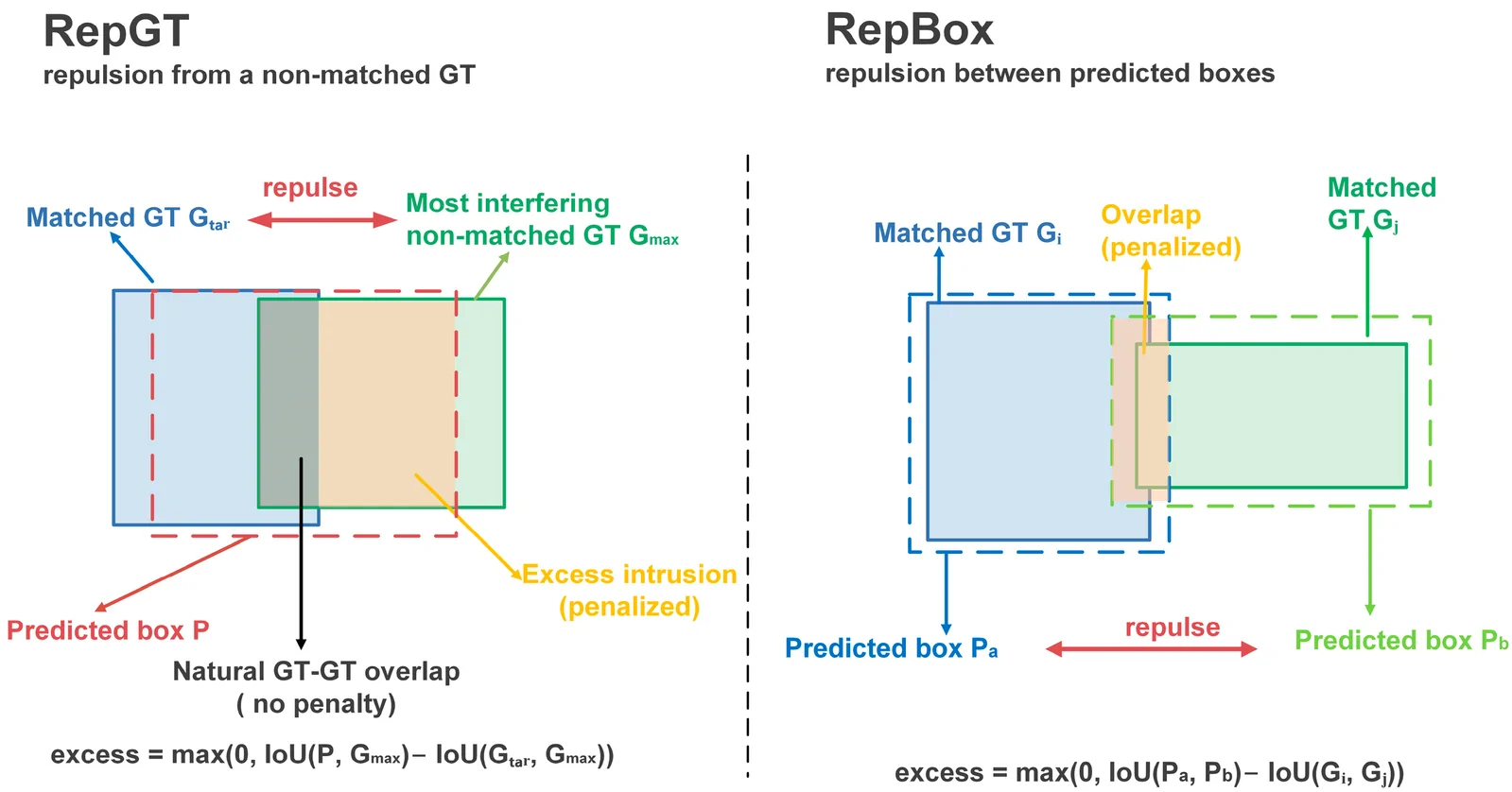
Mechanism of the repulsion loss.

This paper introduces a repulsion loss to compensate for this deficiency. The loss constrains the spatial relationships among predicted boxes: it pushes each predicted box away from non-corresponding ground-truth boxes and other predicted boxes, reducing missed detections of adjacent ground-truth boxes and the suppression of competing detections by NMS, thereby improving detection accuracy in overlapping scenarios. During training, each positive sample predicted box is matched with its ground-truth box through label assignment, and the repulsion loss imposes constraints on two types of spatial interference: the intrusion of a predicted box into a non-corresponding ground-truth box, and the overlap between different predicted boxes. Accordingly, the repulsion loss consists of two terms: the repulsion term between a predicted box and a non-corresponding ground-truth box (RepGT), and the repulsion term between a predicted box and a non-corresponding predicted box (RepBox),and the Figure 3 shows the mechanism of the repulsion loss.

The repulsion term between a predicted box and a non-corresponding ground-truth box (RepGT) forces each positive sample predicted box to stay away from the ground-truth boxes other than its matched ground-truth box. Let $P^{+}$ be the set of positive sample predicted boxes, and let the matched ground-truth box corresponding to $P\in P^{+}$ be $G_{tar}$. Its most interfering non-corresponding ground-truth box $G_{max}$ is defined as the ground-truth box with the largest IoU with P:

(9) $$G_{max}={\mathrm{argmax}}_{G\in G\backslash \left\{G_{tar}\right\}}\mathrm{IoU}\left(PG\right)$$

In overlapping scenarios, ground-truth boxes themselves may overlap each other; penalizing all intrusions would suppress legitimate overlapping detection results. Compared with the original IoG-based RepGT, our formulation quantifies the intrusion with the IoU metric and subtracts the natural overlap degree between the matched and interfering ground-truth boxes, so that legitimate overlap between cochannel signals is not suppressed, because this subtracted term vanishes whenever the two ground-truth boxes do not overlap; the gate is inactive in those cases, and the term reduces to an ungated IoU-based repulsion. Therefore, RepGT only penalizes the part where the intrusion of a predicted box into $G_{max}$ exceeds the natural overlap degree between the ground-truth boxes, and defines the IoU excess:

(10) $$e\left(P\right)=\max\left(0,\mathrm{IoU}\left(P,G_{max}\right)\;-\;\mathrm{IoU}\left(G_{tar},G_{max}\right)\right)$$

Although IoU is not invariant to box enlargement, the proposed RepGT is gated and penalizes only the excess beyond the natural GT overlap. Once the prediction approaches its matched GT, further enlargement is constrained by CIoU and DFL. The single-variable comparison in Section 5.5 shows a small overall gain and essentially unchanged high-overlap performance, so the result is presented as a geometry-aware correction rather than as proof of high-overlap superiority.

After the smooth log function transformation and normalization by the number of positive samples, RepGT is defined as:

(11) $$L_{RepGT}=\left(\frac{1}{\left|P^{+}\right|}\right)\sum_{P\in P^{+}}{\mathrm{Smooth}}_{\ln}(e(P))$$

${\mathrm{Smooth}}_{\ln}$ is a continuously differentiable smooth log function:

(12) $${\mathrm{Smooth}}_{\ln}(x)=\left\{\begin{array}{cc} -\ln(1-x), & x\leq \sigma \\ -\ln(1-\sigma )+\frac{x-\sigma }{1-\sigma }, & x>\sigma \end{array}\right.$$

Here, σ=0.1.

The repulsion term between a predicted box and a non-corresponding predicted box (RepBox) imposes constraints on the overlap between different predicted boxes. In overlapping scenarios, multiple signals may belong to the same modulation category; if the repulsion objects are divided by the predicted category, the overlap between predicted boxes of signals of the same category cannot be constrained. To this end, this paper groups predicted boxes by their matched ground-truth boxes and imposes repulsion constraints on pairs of predicted boxes with different matched ground-truth boxes, so that NMS does not suppress one of them as a duplicate detection in the post-processing stage. Let $P_{a}$ and $P_{b}$ be two predicted boxes whose matched ground-truth boxes are $G_{i}$ and $G_{j}$$\left(G_{i}\neq G_{j}\right)$, respectively, and define the IoU excess of the predicted box pair:

(13) $$e\left(P_{a},P_{b}\right)=\max\left(0,\mathrm{IoU}\left(P_{a},P_{b}\right)-\mathrm{IoU}\left(G_{i},G_{j}\right)\right)$$

That is, the IoU between the predicted boxes minus the natural IoU between their matched ground-truth boxes. After the smooth log function transformation and normalization by the number of valid predicted box pairs, RepBox is defined as:

(14) $$L_{RepBox}=\left(\frac{1}{N_{pairs}}\right)\sum_{a,b}{\mathrm{Smooth}}_{\ln}(e(P_{a},P_{b}))$$

Here, $N_{pairs}$ is the number of predicted-box pairs whose IoU excess is greater than 0, accumulated over the batch. Pairs are formed only between predicted boxes matched to different ground-truth boxes, so $N_{pairs}$ = 0 whenever the positives in a batch are matched to fewer than two ground-truth boxes, or whenever no cross-pair exceeds the natural overlap of its two ground-truth boxes. In that case, the numerator is also zero and $L_{\mathrm{RepBox}}$ is defined as 0, which produces no gradient and leaves the remaining loss terms unchanged. The term is therefore equivalent to a masked mean over valid pairs with max($N_{pairs}$, 1) in the denominator. Because the penalty is accumulated over the batch and divided once by the batch-wide pair count, $L_{\mathrm{RepBox}}$ is a pair-weighted mean rather than an image-wise average, so images containing more valid pairs contribute proportionally more.

RepGT and RepBox address two different failure modes: RepGT suppresses the intrusion of a predicted box into non-matched ground-truth boxes, while RepBox suppresses the mutual overlap between predicted boxes matched to different ground-truth boxes. Since overlapping scenarios exhibit both types of interference, the two terms are used jointly in the complete configuration.

Combining the above basic detection loss and the repulsion loss, the total loss function in the fine-tuning stage is:

(15) $$L_{total}=7.5L_{box}+0.5L_{cls}+1.5L_{DFL}+\lambda_{eff}(L_{\mathrm{RepGT}}+L_{\mathrm{RepBox}})$$

Here, $\lambda_{eff}$ is the effective weight of the repulsion loss. The base detection loss follows the native YOLOv8 gains: 7.5 for box regression, 0.5 for classification, and 1.5 for DFL. RepGT and RepBox are added with equal weight and are therefore on the same scale as the weighted base loss. During warm-up, $\lambda_{eff}$ increases linearly from 0 to its target value and then remains constant.

## 5. Experiments and Results

This section presents a comprehensive experimental validation of the proposed overlapping-signal detection method. The dataset, implementation details, and evaluation metrics are introduced in Section 5.1, Section 5.2 and Section 5.3. Section 5.4 compares the complete method with mainstream detection methods, Section 5.5 verifies the contributions of SimMIM self-supervised pretraining and the repulsion loss through ablation experiments, Section 5.6 reports the initialization control experiment, and Section 5.7 reports the held-out evaluation.

### 5.1. Dataset

The downstream detection dataset is constructed with the WBSig53 framework (TorchSig 2.1.1) [5], with generation seed 42. The construction pipeline is as follows: 3–5 signal targets are first randomly selected from the WBSig53 standard modulation set, covering six classes of digital modulation—amplitude shift keying (ASK), frequency shift keying (FSK), orthogonal frequency-division multiplexing (OFDM), pulse amplitude modulation (PAM), phase shift keying (PSK) and quadrature amplitude modulation (QAM); the center frequency, bandwidth, duration and signal-to-noise ratio (SNR, distributed over [0, 30] dB) of each target are then set independently, and the targets are synthesized into a wideband complex sampling sequence at a sampling rate of 10 MHz; time–frequency images of 512 × 512 are generated by the Short-Time Fourier Transform (STFT) with a Blackman window and no frame overlap, and rendered with the COLORMAP_JET scheme. All optional channel and receiver-impairment transforms of the framework were disabled (impairment level 0), so no Rayleigh fading, frequency-selective multipath, frequency offset, or hardware impairment was applied, and AWGN was the only perturbation; the benchmark therefore isolates signal overlap and additive noise, and no robustness claim is made for the disabled impairments.

Because the signals are synthesized, their ground-truth boxes are obtained analytically rather than by manual annotation: for each target the box is computed from its own generation parameters—start time, duration, bandwidth and center frequency—by the metadata transform of the framework, and the same parameters are written into the COCO-format annotation file together with the class label. The training set contains 5600 images with 23,432 annotated boxes, and the validation set contains 1400 images with 5995 annotated boxes, with an average of 4.184 targets per image in the training set. These 7000 images constitute the downstream control split used for training and model selection. The final evaluation is performed on held-out data that were never used for training, checkpoint selection, or hyperparameter tuning: a 3302-image held-out main set and, drawn from it, a 978-image overlap-stress subset (Section 5.7). SimMIM pretraining uses a separate corpus of 21,250 unlabeled images. Detailed dataset and STFT parameters are listed in Table 1.

**Figure 4:**
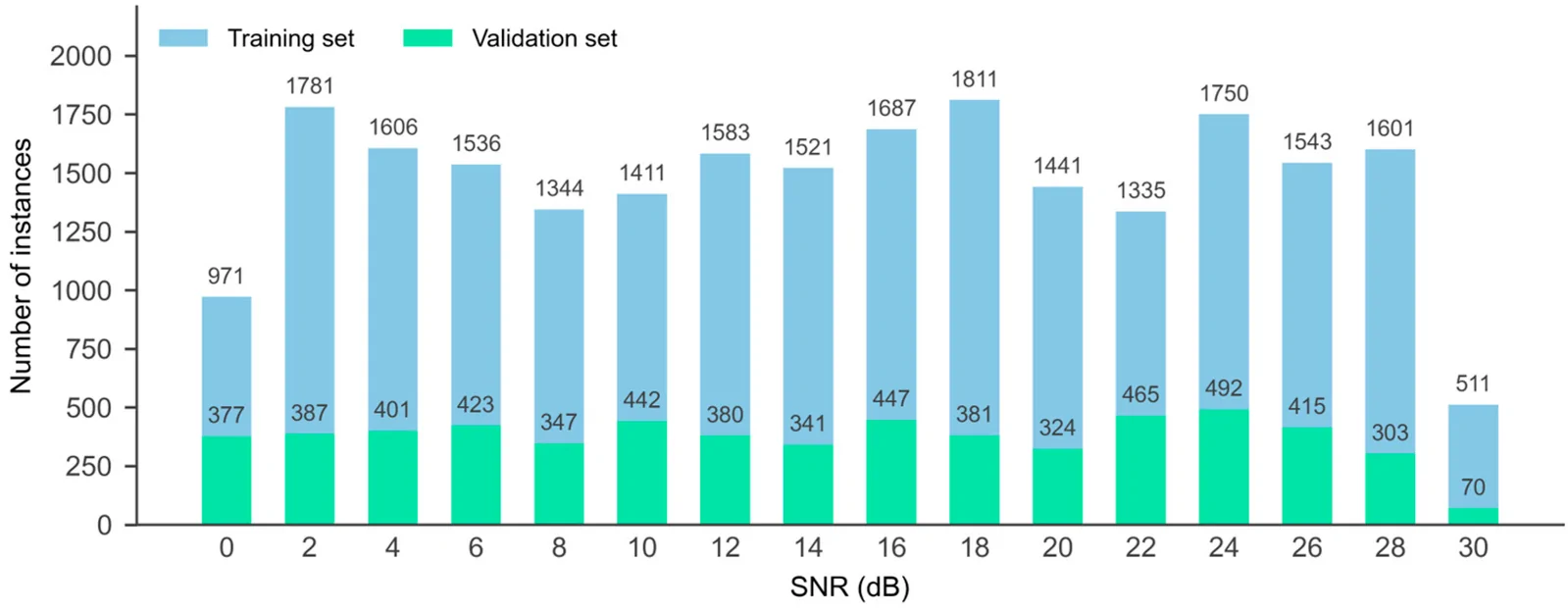
Distribution of instance numbers across SNR levels in the training and validation sets.

To characterize the distribution of the dataset, we count the number of instances in the training and validation sets as a function of SNR, as shown in Figure 4. The instance counts in different SNR bins are relatively uniform, and the validation-set distribution closely matches that of the training set, confirming that the two sets share a similar data composition.

Overlapping scenarios are the most challenging part of the dataset. During generation, each newly added signal has a 30% probability (cochannel overlap probability) of overlapping the signals already placed on the time–frequency plane. Statistics show that 17.7% of the training images and 17.6% of the validation images contain mutually overlapping signal time–frequency regions. In such images, the regions of multiple signals cover each other on the time–frequency plane, and the boundaries of their matched ground-truth boxes are close to or partially coincident with each other, making it difficult for the detector to separate adjacent signal regions.

**Figure 5:**
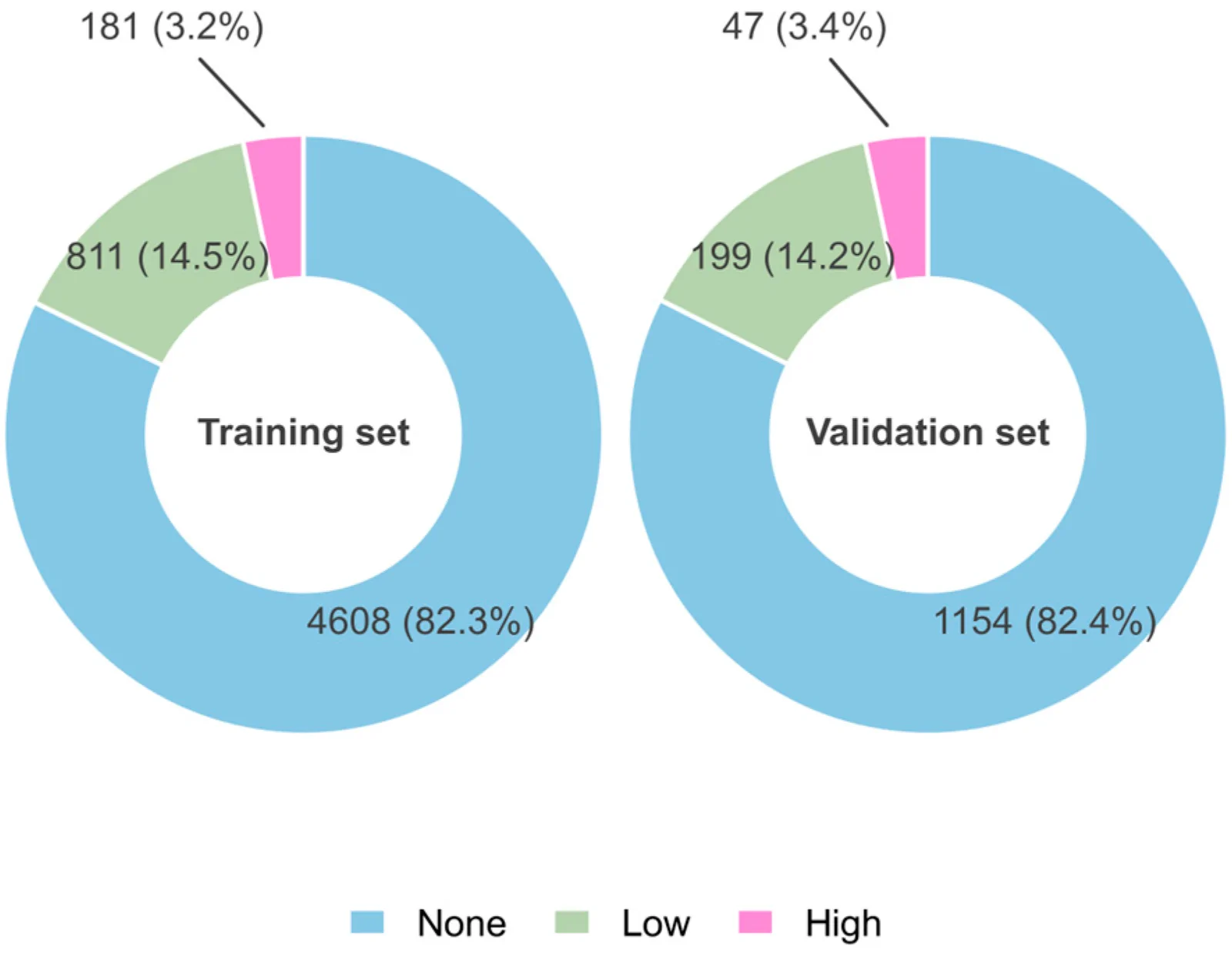
Numbers of images across overlap levels in the training and validation sets.

To analyze the impact of overlap severity on detection performance, images are divided into three overlap levels by the maximum pairwise IoU between the ground-truth boxes of different signals: images without any overlapping pair are assigned to the no-overlap tier, those with a maximum IoU below 0.3 to the low-overlap tier, and those with a maximum IoU of 0.3 or above to the high-overlap tier. In the original 5600-image downstream training subset, the no-, low-, and high-overlap tiers contain 4608 (82.3%), 811 (14.5%), and 181 (3.2%) images, respectively; the corresponding 1400-image validation subset contains 1154 (82.4%), 199 (14.2%), and 47 (3.4%), as shown in Figure 5. The threshold of 0.3 preserves a sufficient number of samples in each tier for tier-wise analysis, while the high-overlap validation subset remains limited to 47 images.

SimMIM self-supervised pretraining uses unlabeled time–frequency images generated with the WBSig53 framework (TorchSig 0.5.3.1), 21,250 in total. The pretraining corpus was generated with the same impairment settings as the downstream corpus: all optional channel and receiver-impairment transforms were disabled. The downstream data use TorchSig 2.1.1, and the control split comprises 5600 training and 1400 validation images. A full MD5 comparison of the 21,250 pretraining images against all downstream images returns an empty intersection, so no identical image file is shared. This is an exact file-level deduplication check; no additional perceptual near-duplicate claim is made. The pretraining data share the same domain as the downstream dataset: COLORMAP_JET pseudo-color rendering, 512 × 512 resolution, six digital modulation classes, and an SNR range of [0, 30] dB; the signal-density range of 1–28 covers the downstream range of 3–5, so the structural diversity of the pretraining data covers the downstream task.

### 5.2. Implementation Details

The proposed method consists of two stages, SimMIM pretraining and downstream detection, both based on PyTorch 2.1 and CUDA 12.1: the pretraining is performed with the MMPretrain framework, and the downstream detection is implemented with the Ultralytics framework (YOLOv8 detection architecture with the backbone replaced by Swin-Tiny). All experiments are conducted on a single NVIDIA RTX 4090 Laptop GPU (16 GB).

SimMIM pretraining is performed on the 21,250 unlabeled time–frequency images for 100 epochs with the AdamW optimizer (learning rate 2 × 10^−4^), a batch size of 32, and automatic mixed precision. In each training iteration, 60% of the image patches are randomly masked with a patch size of 32 × 32, and the network is trained to minimize the L1 loss between the reconstructed and original pixels of the masked patches. After pretraining, the backbone weights are transferred to the detection network at a loading rate of 99.7%, with only the classification head and the dynamic mask buffers skipped.

Downstream fine-tuning is performed on the detection dataset at a unified input size of 640 × 640 for 100 epochs with the SGD optimizer (momentum 0.937, weight decay 0.0005), an initial learning rate of 0.01, and a batch size of 4, preceded by a 3-epoch linear warm-up. Data augmentation consists of HSV color jitter, translation, scaling, horizontal flipping, and Mosaic. These settings are identical across all compared configurations. The hue perturbation is approximately ±2.7° and does not alter time–frequency geometry, labels, or signal placement. COLORMAP_JET is a common pseudo-color mapping in wideband signal detection, and practical systems often store and process spectrograms in this RGB format rather than as grayscale; we retain it to better match practical deployment. A no-hue ablation is left as future work. The repulsion-loss weight follows a dedicated warm-up schedule: it stays at zero during the first 20 epochs, rises linearly to 0.1 over epochs 21–30, and remains constant afterward. All downstream training runs reported in this paper use random seed 42. Experiment-specific settings are reported in the corresponding subsections.

### 5.3. Evaluation Metrics

Evaluation metrics are computed with pycocotools(v2.0.11), with mAP50 and mAP50-95 as the primary metrics. mAP50-95 averages over IoU thresholds in [0.5, 0.95] and is more sensitive to localization errors, capturing the difficulty of bounding-box regression in overlapping scenarios; mAP50 aligns with the commonly used comparison protocol, enabling fair comparison with existing methods. Because missed detections caused by overlapping signals are the most typical failure mode of this task, we additionally report the mean average recall (mAR) as a recall-oriented complement to the average precision. mAR is averaged over the same IoU thresholds and detection-count limits as mAP; it is therefore related to, but not a direct measure of, the missed-detection rate, since it does not penalize duplicate or false detections and does not correspond to a single operating threshold. We further report the number of model parameters (Params) and the floating-point operations (FLOPs) to characterize model complexity. FLOPs are measured on the forward pass with a 640 × 640 input using the same measurement protocol for all compared systems, and parameters are counted with the six object classes. We further report per-class AP50 to observe the performance differences among the six modulation classes. End-to-end inference throughput (FPS) was measured on an NVIDIA RTX 4090 Laptop GPU (16 GB) at a 640 × 640 input resolution, batch size 1, and fp32 precision. Each model was warmed up for 20 iterations and then timed over 200 iterations. The measurement includes preprocessing and non-maximum suppression (NMS).

To unify the evaluation protocol across frameworks, the detection confidence threshold is 0.05 and the maximum number of detections per image is 100; the Faster R-CNN results are converted to the same protocol.

### 5.4. Comparison with Existing Methods

To comprehensively evaluate the proposed method, we compare it against three representative detection models under the same dataset and evaluation protocol: two recent detection models, YOLOv8-M and RT-DETR [34], as well as the classic detection model Faster R-CNN [35]. Faster R-CNN and RT-DETR are trained with their standard implementations and default hyperparameters until convergence.

**Figure 6:**
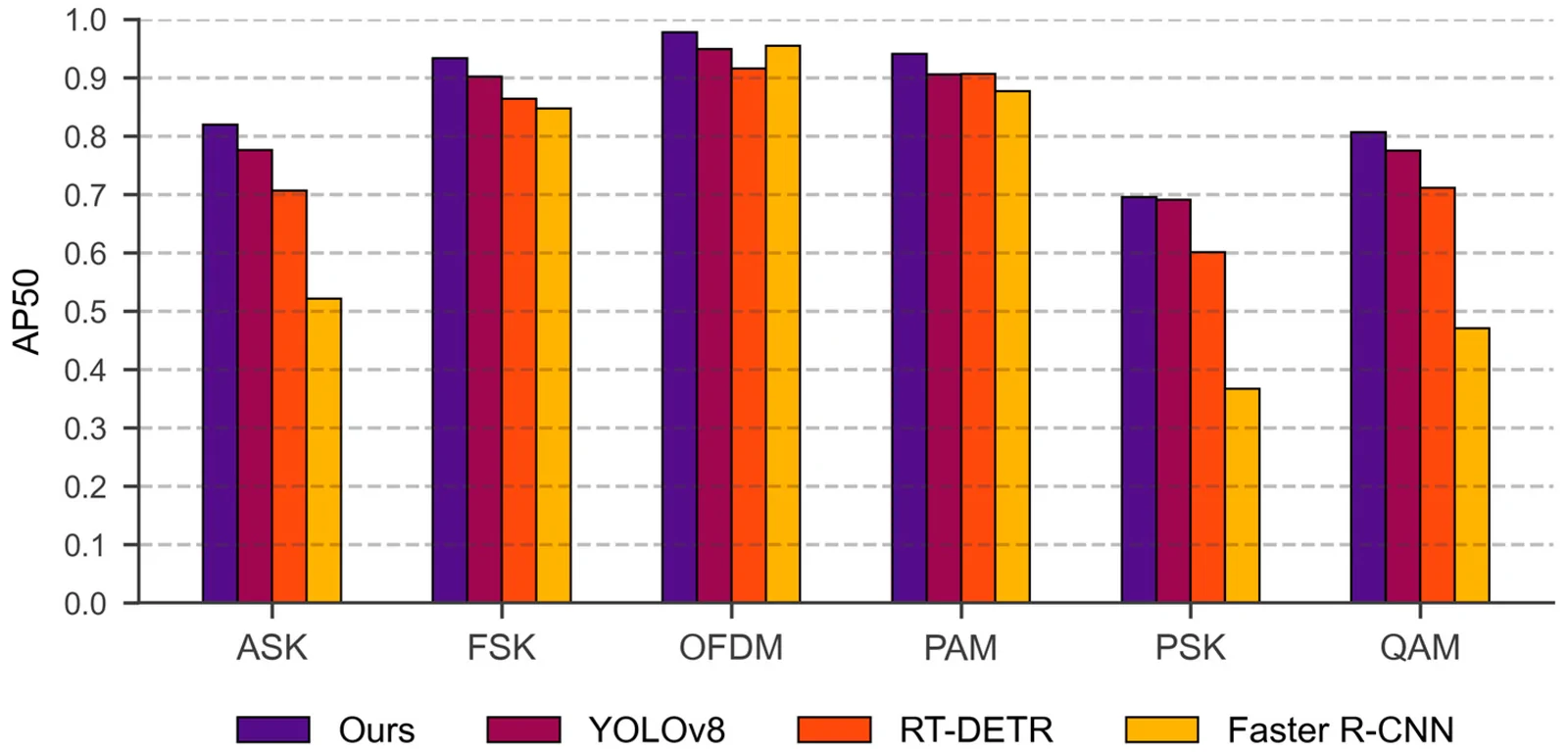
AP50 comparison of different models on the six modulation classes.

As shown in Figure 6, the per-class AP50 of the four models reveals marked differences in difficulty among the six classes. FSK, OFDM, and PAM have clearly distinct time–frequency structures and are detected with relatively high AP50 by all models. In contrast, ASK, PSK, and QAM share similar features within their signal time–frequency regions and are generally harder. Among them, OFDM is the easiest class and PSK the most difficult for all four models. Only our method and Faster R-CNN bring OFDM above 0.95. Our complete method achieves the highest AP50 on all six classes, at 0.820, 0.934, 0.978, and 0.941 on ASK, FSK, OFDM, and PAM, and 0.806 on QAM; on the most difficult class, PSK, it retains a slight edge over YOLOv8 and outperforms Faster R-CNN and RT-DETR by a large margin. The gaps among models widen on the hard classes, where Faster R-CNN drops sharply on ASK, PSK, and QAM while our method and YOLOv8 degrade more gracefully.

**Figure 7:**
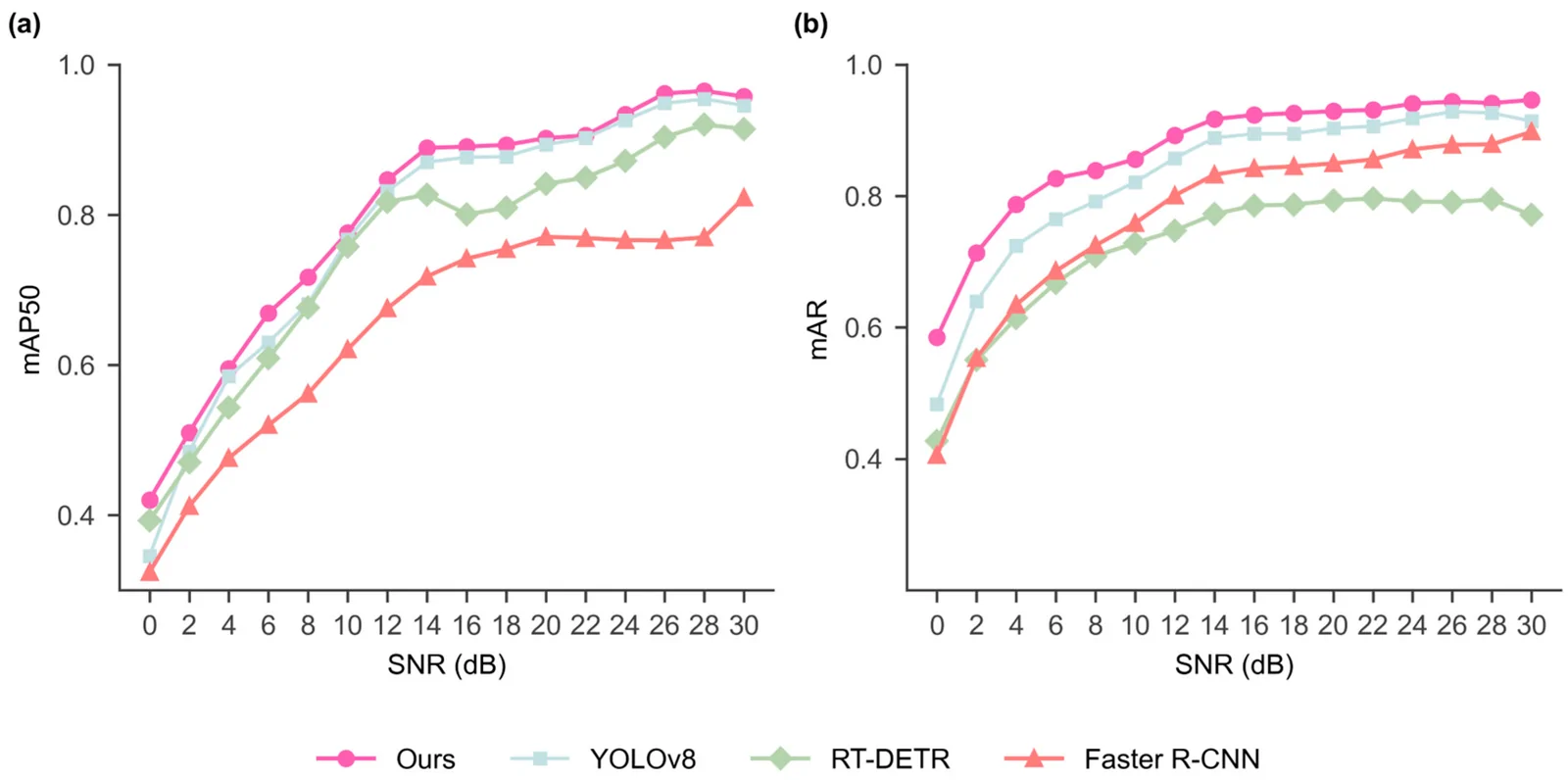
Performance of different models on the validation set versus SNR: (**a**) mAP50; (**b**) mAR.

Figure 7 compares the four models across the SNR range in 2 dB bins, with panel (Figure 7a) reporting mAP50 and panel (Figure 7b) mAR. All models improve with SNR, yet only our method exceeds 0.4 mAP50 in the lowest bin [0, 2) dB, where the three baselines remain below this threshold. In this bin, it achieves 0.420 mAP50 versus 0.346 for YOLOv8 and posts the highest mAR among the four models, 0.585. Above about 8 dB, the two mAP50 curves stay within 0.03 of each other, although our method remains slightly ahead in every bin. Our method outperforms Faster R-CNN in every bin, and the margin grows with SNR to 0.20 at [28, 30) dB. Against RT-DETR, it likewise leads in every bin, with the largest margin of 0.09 at [16, 18) dB. Panel (Figure 7b) shows a clearer advantage: our method achieves the highest mAR in every bin. The margin over YOLOv8 peaks at 0.101 in [0, 2) dB and narrows to about 0.02 at high SNR, where the two curves reach 0.95 and 0.92. RT-DETR and Faster R-CNN trail by 0.05–0.18 across the SNR range, with the largest gaps at low SNR.

**Figure 8:**
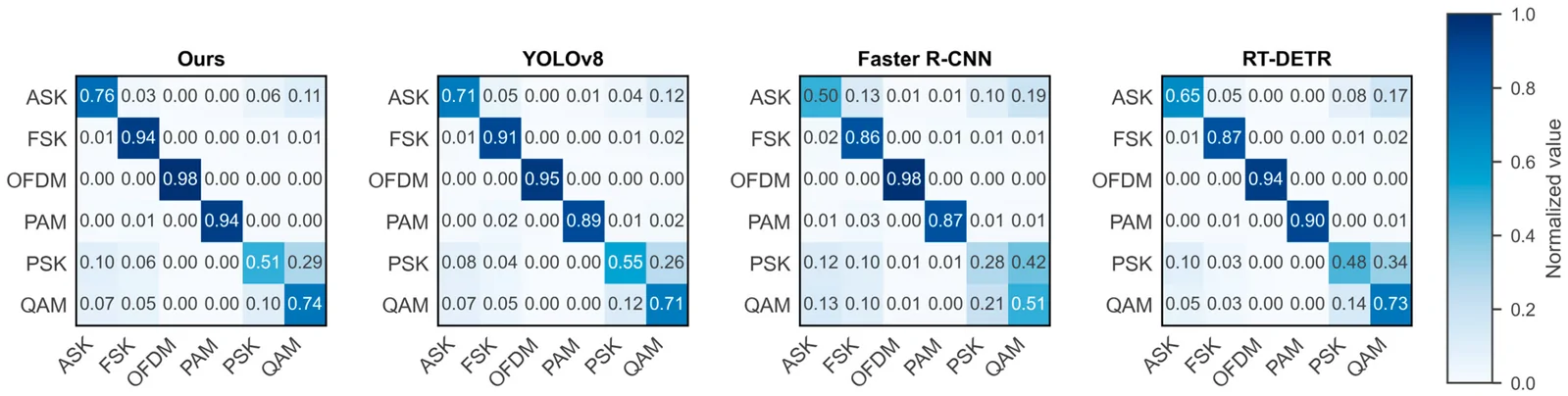
Normalized confusion matrices of the models (Rows are normalized by the number of ground-truth instances of each class; missed detections are excluded, hence row sums are below 1).

Figure 8 presents the normalized confusion matrices of the four models. Misclassification is concentrated among ASK, PSK, and QAM, where PSK→QAM is the most frequent error of every model. In our method, the diagonal entries of FSK, OFDM, and PAM all exceed 0.9, attaining 0.939, 0.981, and 0.935, the highest values among the four models for FSK and PAM. It also holds the highest diagonal on ASK (0.764) and QAM (0.737). YOLOv8 exceeds 0.9 on FSK and OFDM as well. On PSK, the hardest class, our method and YOLOv8 both stay below 0.6. Faster R-CNN degrades most on the hard classes, dropping to just 0.278 on PSK, slightly more than half of our method’s accuracy. RT-DETR has the lowest OFDM accuracy among the four models, and nearly half of its false detections fall on FSK.

Table 2 summarizes the overall performance of the four models on the validation set. On this validation set, our method obtains an mAP50 of 0.862, an mAP50-95 of 0.778, and an mAR of 0.866. Compared with YOLOv8, it improves mAP50, mAP50-95, and mAR by 2.9, 3.7, and 4.2 percentage points, respectively. These values are validation results used for model development and selection; held-out test evidence, which also includes overall performance, is reported separately in Section 5.7. The proposed detector uses 43.1 M parameters and 95.7 GFLOPs at a 640 × 640 input. Its measured end-to-end inference throughput is 50.6 FPS on an NVIDIA RTX 4090 Laptop GPU, compared with 72.9 FPS for YOLOv8 and 47.0 FPS for RT-DETR. The proposed detector is computationally lighter than RT-DETR (110 GFLOPs) and Faster R-CNN (134 GFLOPs), but heavier than YOLOv8 (79.1 GFLOPs); consistently, its throughput of 50.6 FPS exceeds that of RT-DETR (47.0 FPS) while remaining below that of YOLOv8 (72.9 FPS). The accuracy gains reported here are therefore obtained at a lower computational cost than the two heavier baselines, whereas against YOLOv8 the advantage of the proposed method lies in detection accuracy on overlapping signals rather than in computational efficiency. The model exceeds 30 FPS and thus supports near-real-time processing on the tested hardware. Its larger advantage is concentrated in low-SNR and high-overlap cases, where the additional computational cost is justified by improved detection accuracy. Against RT-DETR, the improvements reach 7.8 percentage points in mAP50 and 20.1 percentage points in mAP50-95. The widening gap under stricter metrics indicates that the DETR-style detector, trained with Hungarian matching and ImageNet/COCO pretraining, shows lower accuracy on dense overlapping targets in time–frequency images; its recall (mAR 0.719) is even lower than that of the two-stage Faster R-CNN (0.762). Faster R-CNN achieves the second-highest mAR among the baselines (0.762) but the lowest mAP50 (0.673). The two-stage design recalls overlapping instances at the proposal stage yet confuses them at the RoI classification stage, while requiring the largest FLOP count (134G FLOPs) among the compared models. This experiment compares complete detection systems under a common data split, a common input resolution, and the unified evaluation protocol of Section 5.3. Because the four systems differ in backbone family, model capacity, source of initialization, and detection paradigm, the comparison is system-level only: it does not isolate the contribution of any single component, and no capacity-matched or recipe-matched claim is drawn from it. Component-level evidence is reported in the controlled comparisons of Section 5.5 and Section 5.6.

Table 3 reports the validation-set mAP50 of the four models under three overlap levels. Our method drops by 17.3% from the no-overlap to the high-overlap tier, versus 22.2% for YOLOv8 and 22.6% for RT-DETR. Faster R-CNN shows an even smaller drop (16.7%), but from the lowest base of 0.700 without overlap. On this validation set, our method achieves the highest score in every tier, and the paired-bootstrap margins over YOLOv8 are +2.59 points (95% CI [+1.23, +3.95]) on the no-overlap tier, +3.00 points (95% CI [+0.66, +5.53]) on the low-overlap tier and +6.26 points (95% CI [+2.63, +10.02]) on the 47-image high-overlap tier; all three intervals exclude zero. In the high-overlap tier, it stays at 0.736, above the no-overlap score of Faster R-CNN, while YOLOv8 falls to 0.672. RT-DETR ranks third in every tier, trailing our method by 7.1 to 10.2 points. Faster R-CNN performs worst, recording the lowest score in every tier. All bootstrap intervals reported in this paper are obtained by image-level paired bootstrap with 2000 resamples and random seed 42: the resampling unit is the image rather than the box, and both arms are evaluated on the same resampled image indices. Because the interval quantifies evaluation-set sampling uncertainty, we do not present it as a substitute for repeated-seed statistics.

**Figure 9:**
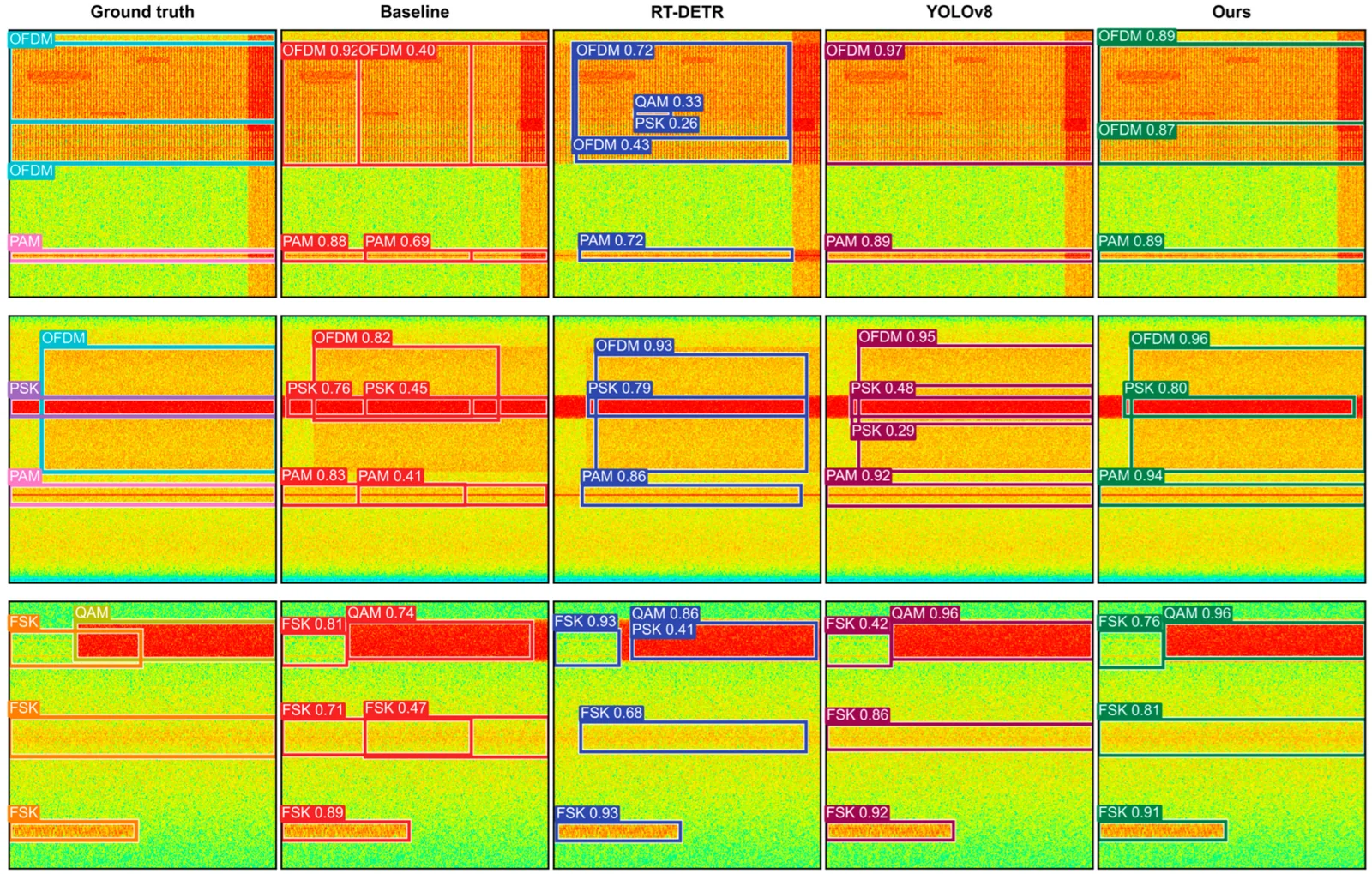
Visualization of detection results.

To provide a more intuitive view of these differences, Figure 9 presents detection examples from three representative scenes covering the low- and high-overlap tiers. Each row corresponds to one scene and shows, from left to right, the ground-truth boxes and the predictions of baseline RT-DETR, YOLOv8, and the proposed method.

### 5.5. Ablation Experiments

An ablation study was conducted to evaluate the contributions of SimMIM self-supervised pretraining and the repulsion loss, the two core components of the proposed method. The study followed a 2 × 2 design with the backbone initialization and the repulsion loss as the two factors. The four configurations are the baseline, the pretraining-only configuration, the repulsion-only configuration, and the complete method. The complete method combines both components, while the baseline uses a randomly initialized backbone without the repulsion loss.

Table 4 summarizes the detection performance of the four configurations. Relative to the baseline, the pretraining-only configuration improves mAP50 by 6.1 points and mAP50-95 by 6.6 points. The repulsion-only configuration yields similar gains of 5.4 and 5.5 points, respectively. These configuration-level results show that both components contribute to the complete pipeline, but the pretraining-only gain should not be read as the isolated effect of SimMIM. Meanwhile, SimMIM pretraining improves mAP50-95 more than mAP50, whereas the repulsion loss improves both metrics to a similar degree. When the two components are combined, the complete configuration improves mAP50 by 9.0 points and mAP50-95 by 9.8 points over the baseline. The combined gain, however, narrows markedly compared with the sum of the individual gains. Adding the repulsion loss to the pretraining-only configuration improves mAP50 by only 3.0 points and mAP50-95 by 3.2 points. In contrast, adding SimMIM pretraining to the repulsion-only configuration improves mAP50 by 3.7 points and mAP50-95 by 4.3 points.

The repulsion loss alone improves mAP50 by 5.4 points, but its benefit varies with the weight $\lambda_{eff}$. To compare the performance under different weights and overlap levels, we trained five models with $\lambda_{eff}$ = 0, 0.1, 0.25, 0.5, and 0.75 on the pretrained backbone. The results are reported in Table 5.

Here, denotes the repulsion-loss weight. The baseline row corresponds to the randomly initialized model trained without SimMIM pretraining and without the repulsion loss; the row is the pretraining-only configuration. Overall performance is non-monotonic across weights and is highest in this comparison (0.862 mAP50). In the high-overlap tier, the complete configuration improves mAP50 from 0.661 for the baseline to 0.736, an observed combined gain of 7.5 points, for which the image-level paired bootstrap gives +6.90 points (95% CI [+1.75, +11.87]). Increasing from 0 to 0.1 gives an additional 5.5-point improvement over the pretraining-only configuration (0.681 to 0.736). Although the no- and low-overlap scores are non-monotonic in the weight, they end lower at 0.75 (0.836 and 0.724) than at 0.1 (0.890 and 0.779). These results identify 0.1 as the preferred operating point in this sweep.

To isolate the effect of the modified RepGT, we compared the original IoG and proposed gated IoU-excess formulations under identical data, input size, batch size, learning-rate schedule, and repulsion weight, changing only the mode of RepGT. The two variants obtain mAP50 of 0.8360 and 0.8391, mAP50-95 of 0.7482 and 0.7507, AP75 of 0.8047 and 0.8112, and mAR of 0.8482 and 0.8495. Throughout this comparison, differences are reported as gated minus original, so that a negative value favors the original IoG formulation. At high overlap, the two formulations are 0.6823 and 0.6811, a gated-minus-original difference of −0.12 points (paired bootstrap −0.29 points, 95% CI [−3.82, +3.12]); on the held-out high-overlap subset defined in Section 5.7 (74 images), the same contrast is −1.56 points (95% CI [−3.44, +0.44]). Both intervals include zero, so the high-overlap difference is reported as descriptive. The modified formulation is better on all four overall metrics, while the high-overlap difference is small and falls within bootstrap uncertainty. This is consistent with a definitional correction rather than a performance improvement, and we therefore draw no high-overlap conclusion from this comparison. This gating is not a vacuous correction. On the downstream corpus, 736 of the 5995 ground-truth boxes in the validation split (12.3%) and 3136 of 23,432 (i.e., 13.4%) in the training split have at least one neighboring ground-truth box with a non-zero IoU, with a median natural overlap of 0.063 (interquartile range 0.016–0.157, maximum 0.93). On those pairs, the original term penalizes the entire intrusion, although up to that amount is legitimate coexistence, whereas the modified term penalizes only the excess. The correction is therefore designed to be neutral with respect to detection performance and to remove a definitional artifact of the original term; consistently, the observed high-overlap difference is small and slightly negative and lies within bootstrap uncertainty. We therefore retain the modification as a task-specific correction assessed within the complete framework, and do not claim a high-overlap advantage.

### 5.6. Control Experiment

To examine the contribution of initialization, we trained four Swin-Tiny detectors with the same neck and YOLOv8 detection head, 640 × 640 input, and the same 5600-image training and 1400-image validation control split. Only the source of backbone initialization differed: random initialization, ImageNet-1K initialization, an MAE-style masked-reconstruction model, and the proposed SimMIM-Tiny model.

Table 6 reports the comparison across the four initialization settings. The mAP50 values are 0.772, 0.8042, 0.8247, and 0.833, the mAP50-95 values are 0.6800, 0.7213, 0.7337, and 0.746, and the high-overlap mAP50 values are 0.6605, 0.663, 0.677, and 0.681. All three metrics increase monotonically from random initialization to ImageNet-1K, MAE-style, and SimMIM initialization. Relative to random initialization, ImageNet-1K, MAE-style, and SimMIM initialization improve mAP50 by 3.22, 5.27, and 6.10 points, mAP50-95 by 4.13, 5.37, and 6.60 points, and high-overlap mAP50 by 0.25, 1.65, and 2.05 points, respectively; read as successive steps, each transition adds 3.22, 2.05, and 0.83 points of mAP50, 4.13, 1.24, and 1.23 points of mAP50-95, and 0.25, 1.40, and 0.40 points of high-overlap mAP50. Because the backbone and the detection head are identical in all four rows, any difference between them can be attributed to the backbone initialization alone. Within this control, all three metrics increase monotonically from random initialization through ImageNet-1K and MAE-style to SimMIM, and both self-supervised initializations outperform the generic ImageNet-1K initialization. The two initializations also differ in the labeled data they rely on: the ImageNet-1K initialization is derived from 1.28 million labeled images, whereas the SimMIM initialization uses 21,250 unlabeled in-domain images; under this otherwise fixed configuration, the in-domain unlabeled initialization is ahead by 2.88 points of mAP50. In this sense, in-domain self-supervised pretraining substitutes unlabeled in-domain data for a large labeled corpus. This control is an initialization-only comparison under fixed architecture and capacity. The MAE-style baseline uses an eight-layer convolutional decoder, while SimMIM adopts a single-layer linear decoder. The comparison therefore involves two complete pretraining recipes, not a single isolated loss term.

### 5.7. Held-Out Evaluation

This section reports the held-out evidence. The main held-out set contains 3302 images. The overlap-stress subset consists of 978 of these images: all 489 main-set images with a non-zero maximum pairwise ground-truth IoU, together with 489 images whose maximum IoU is zero. Both were generated with the same pipeline as the control split but were never used for training, checkpoint selection, or hyperparameter tuning. The high-overlap subset reported here consists of the 74 main-set images whose maximum pairwise ground-truth IoU is at least 0.30. Section 5.5 and Section 5.6 are computed on the 1400-image control split used for model selection, whereas the results below are computed on the held-out sets.

Table 7 reports the held-out contrasts. The first group compares complete systems that differ in several factors at once, whereas the second group contains single-variable contrasts in which only one component differs.

Table 7 shows that the complete framework exceeds the randomly initialized baseline by 8.24 points of mAP50 on the main held-out set and by 8.43 points on the overlap-stress set, and the YOLOv8 baseline by 1.95 and 2.18 points. Both sets were withheld from training and model selection, and the margins are no smaller on the stress set, so the advantage is concentrated in the more heavily overlapped conditions. The mAP50-95 margins exceed the mAP50 margins (9.10 versus 8.24 points and 3.20 versus 1.95 points), indicating that the framework improves localization at stricter IoU thresholds rather than detection confidence alone. The component-level contrasts are smaller, and the largest of them comes from the repulsion-loss configuration, at 2.57 and 2.50 points of mAP50. The two pretraining recipes differ by less than one point of mAP50 on the main set and by 1.12 points on the stress set.

Table 8 reports the absolute performance of the two methods on the same three held-out settings. The complete framework leads the YOLOv8 baseline in mAP50, mAP50-95, and mAR on the main and stress sets. Its mAP50 margin grows from 1.95 points on the main set to 2.18 points on the overlap-stress set, so the advantage widens as the overlap load increases. Both methods score about 12 points lower on the 74-image high-overlap subset than on the main set (0.7543 and 0.7379 against 0.8750 and 0.8555), so that subset is the harder regime for both, and the framework remains ahead there by 1.64 points. Its mAR is also higher on both larger sets, 0.8731 and 0.8289 against 0.8437 and 0.7935, so the mAP50 gain is accompanied by a higher recall rather than traded against it.

Taken together, the two tables show that the framework’s held-out margins are largest over its own randomly initialized baseline and smaller but consistent over the YOLOv8 baseline, and that neither margin shrinks under overlap stress, so the advantage holds on data that were never used for training or for model selection. The margins also survive the hardest subset available, the 74 images with the highest overlap, although that subset is small enough to support only a descriptive reading. The component-level contrasts are appreciably smaller than the framework-level ones, so the held-out evidence supports the complete framework, and the contribution of the individual components is assessed in the controlled comparisons of Section 5.5 and Section 5.6.

## 6. Discussion

The configuration-level ablation shows that both components contribute: SimMIM pretraining and the repulsion loss each improve the randomly initialized baseline. Their combination, however, is sub-additive: on the validation set it improves mAP50 by 9.0 points over that baseline, less than the 11.5 points expected from the sum of the individual gains. The two components contribute unevenly across overlap tiers. In the no- and low-overlap tiers, the pretraining-only configuration reaches 0.863 and 0.746 mAP50, close to the 0.890 and 0.779 of the complete framework. The repulsion loss therefore adds comparatively little in these tiers. In the high-overlap tier, by contrast, the baseline reaches 0.661 mAP50 and the complete framework 0.736, a combined gain of 7.5 points, whereas the pretraining-only configuration reaches only 0.681. Raising $\lambda_{eff}$ from 0 to 0.1 therefore adds 5.5 points at high overlap, which is the incremental contribution of the repulsion loss after pretraining. We attribute this pattern to feature mixing: mixed targets are difficult to distinguish, which in turn makes it difficult for the repulsion loss to determine which predicted boxes should repel each other. The two components therefore act in sequence: pretraining first separates the targets in the feature representation, and the repulsion loss is effective only once it has done so. The sensitivity analysis is consistent with this account: after pretraining, a weight as small as 0.1 already performs best. A weight of 0.75 falls below the pretraining-only level in overall mAP50 (0.807 versus 0.833).

Compared with YOLOv8, the advantage of the proposed framework grows as the detection task becomes harder. At the lowest SNR bin, the observed lead reaches 7.4 points in mAP50, and on the validation high-overlap tier it reaches 6.4 points, for which the paired-bootstrap estimate is 6.26 points (95% CI [+2.63, +10.02]). In terms of recall, the proposed framework remains at least on par with YOLOv8 across all SNR bins, although the mAP50 curves are close in the mid-to-high SNR range. Three mechanisms plausibly explain this pattern. First, the Swin-Transformer backbone establishes cross-window long-range interactions through shifted-window self-attention, whose global receptive field helps relate weak signals to their surrounding context at low SNR. Second, SimMIM pretraining produces in-domain feature representations that preserve the global time–frequency structure, which the shifted-window attention of the Swin backbone can exploit across progressively larger receptive fields. Third, the repulsion loss acts directly on the interference between overlapping targets, which is most useful in the high-overlap cases. The margin is concentrated in the difficult regimes rather than being uniform across all conditions: YOLOv8 remains competitive in simpler mid-to-high SNR cases, whereas the proposed framework provides larger gains under low-SNR and high-overlap interference. The equal weighting of RepGT and RepBox may also limit the repulsion loss, since the two terms address different failure modes and may require different weights.

The above analysis also points to several limitations. The downstream and pretraining corpora are both synthetically generated and limited in scale. Because the annotations are computed from the signal parameters rather than produced by manual labeling, the corpus does not exercise annotation cost, and a labeled-data-ratio study on it would vary the number of training images rather than the annotation effort; we therefore report no such study. Synthetic generation is what makes controlled manipulation of SNR and overlap possible, but the acquisition of captured over-the-air and fading-channel data, and validation on it, is also outside the scope of the present study. The high-overlap subsets are small, so we report high-overlap margins as descriptive evidence, and every high-overlap margin is either reported with an image-level paired bootstrap interval or stated as descriptive; multi-seed repetition of the main configuration was not performed, so the intervals quantify evaluation-set uncertainty rather than run-to-run variability.

## 7. Conclusions

This paper proposed a framework for detecting overlapping modulated signals in time–frequency images. It combines SimMIM self-supervised pretraining of a Swin-Tiny backbone on unlabeled in-domain time–frequency images with a repulsion loss that suppresses interference between overlapping targets. The complete framework improves mAP50 by 9.0 points over the random-initialized baseline on the validation set and retains margins of 1.95 and 2.18 points over the YOLOv8 baseline on the held-out main and overlap-stress sets. On the main held-out set, it obtains 0.875 mAP50, 0.798 mAP50-95, and 0.873 mAR. The advantage is most evident at low SNR and high overlap: the observed mAP50 lead reaches 7.4 points at the lowest SNR bin and 6.4 points on the validation high-overlap tier (paired-bootstrap estimate 6.26 points, 95% CI [+2.63, +10.02]), while performance in the mid-to-high SNR range is comparable to YOLOv8. These results indicate that in-domain self-supervised pretraining combined with spatially constrained box regression offers a practical direction for signal detection in complex electromagnetic environments.

## Figures and Tables

**Table 1:** Parameters of the WBSig53 dataset and STFT.

Parameter	Value
Data form	Complex sampling sequence
Sampling length	262,144
SNR range	[0, 30] dB
FFT points	512
Window function	Blackman
STFT window overlap rate	0
Time–frequency image size	512 × 512
Color scheme	COLORMAP_JET
Modulation classes	ASK, FSK, OFDM, PAM, PSK, QAM
Signals per image	3–5
Channel model	AWGN only
impairments	Disabled (impairment level 0)

**Table 2:** Comparison with existing detection methods on the validation set. Parameters and FLOPs are reported for the complete detector at a 640 × 640 input resolution, measured with the same protocol for all systems; FPS follows the measurement protocol in Section 5.3.

Model	Backbone	mAP50	mAP50-95	mAR	Parameters/FLOPs	FPS
Ours	Swin-Tiny (SimMIM)	0.862	0.778	0.866	43.1 M/95.7 G	50.6
YOLOv8	CSPDarkNet-M (random)	0.833	0.741	0.824	25.9 M/79.1 G	72.9
RT-DETR	HGNetv2-L (COCO)	0.784	0.577	0.719	32 M/110 G	47.0
Faster R-CNN	ResNet-50-FPN (ImageNet)	0.673	0.535	0.762	41.3 M/134 G	-

**Table 3:** mAP50 of different models under different overlap levels.

Model	No Overlap	Low Overlap	High Overlap
Ours	0.890	0.779	0.736
YOLOv8	0.864	0.751	0.672
RT-DETR	0.819	0.694	0.634
Faster R-CNN	0.700	0.603	0.583

**Table 4:** Model ablation experiments.

Model	SimMIM	Repulsion Loss	mAP50	mAP50-95
Baseline	---	---	0.772	0.680
Ours	√	---	0.833	0.746
	---	√	0.826	0.735
	√	√	0.862	0.778

**Table 5:** Detection performance under different repulsion-loss weights and overlap levels.

$\lambda_{eff}$	No Overlap	Low Overlap	High Overlap	mAP50 (Total)
Baseline	0.804	0.689	0.661	0.772
0	0.863	0.746	0.681	0.833
0.1	0.890	0.779	0.736	0.862
0.25	0.841	0.729	0.698	0.811
0.5	0.865	0.749	0.727	0.835
0.75	0.836	0.724	0.704	0.807

**Table 6:** Controlled comparison of backbone initialization under the same Swin-Tiny backbone and YOLOv8 detection head.

Initialization	mAP50	mAP50-95	High-Overlap mAP50
Random	0.772	0.680	0.6605
ImageNet-1K	0.8042	0.7213	0.663
MAE	0.8247	0.7337	0.677
SimMIM	0.833	0.746	0.681

**Table 7:** Contrasts on the enlarged held-out test sets.(ΔmAP50, ΔmAP50-95 and ΔmAR are reported in percentage points, and a positive value favors the first configuration in each contrast.)

Contrast	ΔmAP50 (Main/Stress)	ΔmAP50-95 (Main/Stress)	ΔmAR (Main/Stress)
Complete framework vs. random-initialized baseline	+8.24/+8.43	+9.10/+9.24	+3.84/+4.48
Complete framework vs. YOLOv8	+1.95/+2.18	+3.20/+3.55	+2.95/+3.54
Repulsion loss vs. no repulsion	+2.57/+2.50	+2.75/+2.82	+0.98/+1.24
SimMIM vs. MAE	+0.75/+1.13	+1.10/+1.27	+0.72/+0.58

**Note:** The main held-out set contains 3302 images and the overlap-stress subset contains 978 images. Neither was used for training, checkpoint selection, or hyperparameter tuning.

**Table 8:** Performance of the complete framework and the YOLOv8 baseline on the enlarged held-out test sets.

Test Setting	Complete Framework (mAP50/mAP50-95/mAR)	YOLOv8 (mAP50/mAP50-95/mAR)	ΔmAP50
Main held-out set	0.8750/0.7975/0.8731	0.8555/0.7655/0.8437	+1.95
Overlap stress set	0.8378/0.7562/0.8289	0.8160/0.7207/0.7935	+2.18
Main high overlap subset	0.7543/0.6751/0.7346	0.7379/0.6410/0.7020	+1.64

**Note:** ΔmAP50 is reported in percentage points. All three settings were evaluated under the same protocol (pycocotools, confidence threshold 0.05, NMS IoU 0.7, at most 100 detections per image).

## Data Availability

The data analyzed in this study are synthetic and were generated with the TorchSig framework (TorchSig 2.1.1 for the downstream corpus and TorchSig 0.5.3.1 for the pretraining corpus), using generation seed 42. The cited TorchSig repositories provide the generation framework, not the datasets used here. The generation configuration files, the data-split definition files, the split lists, the COCO-format annotation files, and the scripts that produce the spectrograms and compute the overlap tiers are available from the corresponding author on reasonable request. The downstream corpus comprises 5600 training images and 1400 validation images, which together form the 7000-image control split used for training and model selection, together with a 3302-image held-out main set. The held-out main set is defined by an exclusion rule rather than by sampling: it consists of the generated downstream images that were not assigned to the control split, so no sampling seed is involved for it. The 978-image overlap-stress subset is drawn from the held-out main set: it contains all 489 main-set images whose maximum pairwise ground-truth IoU is greater than zero, together with 489 of the main-set images whose maximum pairwise IoU is zero, drawn at random with seed 42. The pretraining corpus contains 21,250 unlabeled images generated in a separate run. The generation parameters are reported in Section 5.1 and Table 1, including the disabled channel and receiver-impairment transforms. All training runs and all bootstrap resamplings use random seed 42. Spectrograms are computed by a 512-point STFT with a Blackman window and no overlap, rendered as 512 × 512 COLORMAP_JET images and normalized by the infinity norm before a dB mapping onto the 8-bit intensity range. No third-party or human-subject data were used.

## Abbreviations

The following abbreviations are used in this manuscript:

AWGN	additive white Gaussian noise
BCE	binary cross-entropy
CIoU	complete intersection over union
CNN	convolutional neural network
COCO	Common Objects in Context
DETR	detection transformer
DFL	distribution focal loss
DIoU	distance intersection over union
FFT	fast Fourier transform
FLOPs	floating-point operations
FPS	Frames Per Second
GFLOPs	giga floating-point operations
GIoU	generalized intersection over union
HSV	hue, saturation, value
IoG	intersection over ground-truth
IoU	intersection over union
MAE	masked autoencoder
mAP	mean average precision
mAP50	mean average precision at an IoU threshold of 0.50
mAP50-95	mean average precision averaged over IoU thresholds from 0.50 to 0.95
mAR	mean average recall
MIM	masked image modeling
NMS	non-maximum suppression
OFDM	orthogonal frequency-division multiplexing
PAM	pulse amplitude modulation
PAN-FPN	Path Aggregation Network-Feature Pyramid Network
PSK	phase shift keying
QAM	quadrature amplitude modulation
RepBox	predicted-box repulsion term
RepGT	repulsion ground-truth loss
RF	radio frequency
RoI	region of interest
RT-DETR	real-time detection transformer
SGD	stochastic gradient descent
SimMIM	simple masked image modeling
SNR	signal-to-noise ratio
STFT	short-time Fourier transform
WBSig53	WidebandSig53
YOLO	You Only Look Once
YOLOv8	You Only Look Once version 8
